# Prolonged In Vivo Chemogenetic Generation of Hydrogen Peroxide by Endothelial Cells Induces Cardiac Remodelling and Vascular Dysfunction

**DOI:** 10.3390/antiox14060705

**Published:** 2025-06-10

**Authors:** Melina Lopez, Niklas Herrle, Bardia Amirmiran, Pedro F. Malacarne, Julia Werkhäuser, Souradeep Chatterjee, Carine Kader, Victoria Jurisch, Xin Wen, Maedeh Gheisari, Katrin Schäfer, Christian Münch, Florian Leuschner, Ralf Gilsbach, Flávia Rezende, Ralf P. Brandes

**Affiliations:** 1 Institute for Cardiovascular Physiology, Goethe University, 60590 Frankfurt am Main, Germany; mlopez@vrc.uni-frankfurt.de (M.L.); nmueller@vrc.uni-frankfurt.de (N.H.); amirmiran@vrc.uni-frankfurt.de (B.A.); malacarne@vrc.uni-frankfurt.de (P.F.M.); werkhaeuser@vrc.uni-frankfurt.de (J.W.); chatterjee@vrc.uni-frankfurt.de (S.C.); kader@vrc.uni-frankfurt.de (C.K.); jurisch@vrc.uni-frankfurt.de (V.J.); wen@vrc.uni-frankfurt.de (X.W.); gheisari@vrc.uni-frankfurt.de (M.G.); 2 German Centre of Cardiovascular Research (DZHK), Partner Site Rhein Main, 60590 Frankfurt am Main, Germany; 3 Department of Cardiology, University Medical Center Mainz, 55131 Mainz, Germany; katrin.schaefer@unimedizin-mainz.de; 4 Institute of Molecular Systems Medicine, Goethe University, 60438 Frankfurt am Main, Germany; ch.muench@em.uni-frankfurt.de; 5 Department of Cardiology, Angiology and Pulmonology, University Hospital Heidelberg, 69118 Heidelberg, Germany; florian.leuschner@med.uni-heidelberg.de; 6 German Centre of Cardiovascular Research (DZHK), Partner Site Heidelberg/Mannheim, 69120 Heidelberg, Germany; ralf.gilsbach@uni-heidelberg.de; 7 Institute of Experimental Cardiology, Heidelberg University Hospital, 69118 Heidelberg, Germany; 8 Institut für Kardiovaskuläre Physiologie, Fachbereich Medizin der Goethe-Universität, Theodor-Stern Kai 7, 60590 Frankfurt am Main, Germany

**Keywords:** D-amino acid oxidase, chemogenetic, oxidative stress, reactive oxygen species, endothelial cells, myocardial infarction, vascular dysfunction

## Abstract

Increased levels of reactive oxygen species (ROS) are a hallmark of cardiovascular disease. ROS impact the function of proteins largely through thiol modification leading to redox signalling. Acute, targeted interference with local ROS levels has been difficult. Therefore, how dynamics in redox signalling impact cardiovascular health is still a matter of current research. An inducible, endothelial cell-specific knock-in mouse model expressing a yeast D-amino acid oxidase enzyme was generated (Hipp11-Flox-Stop-Flox-yDAO-Cdh5-CreERT2^+/0^ referred to as ecDAO). DAO releases H_2_O_2_ as a by-product of the conversion of D-amino acids into imino acids. The D-amino acid treatment of DAO-expressing cells therefore increases their intracellular H_2_O_2_ production. The induction of yDAO in the ecDAO mice was performed with tamoxifen. Subsequently, the mice received D-Alanine (D-Ala, 0.5 M) through drinking water, and the effects on ROS production and vascular and cardiac function were determined. ecDAO induction increased endothelial ROS production as well as ROS production in the lung, which is rich in endothelial cells. The functional consequences of this were, however limited: After minimally invasive myocardial infarction, there was no difference in the outcome between the control (CTL) and ecDAO mice. With respect to vascular function, three days of D-Ala slightly improved vascular function as demonstrated by an increase in the diameter of the carotid artery in vivo and decreased vessel constriction to phenylephrine. Fifty-two days of D-Ala induced cardiac remodelling, increased peripheral resistance, and overoxidation of peroxiredoxins. In conclusion, acute stimulation of endothelial ROS improves cardiovascular function, whereas prolonged ROS exposure deteriorates it.

## 1. Introduction

Reactive oxygen species (ROS) contribute to the development of and are potential drivers of cardiovascular diseases (CVDs). Atherosclerosis, ischemia/reperfusion, septicemia, heart failure, and myocardial infarction are associated with an increase in oxidative stress caused by high concentrations of ROS. Therefore, ROS are often considered mediators of disease-associated damage and linked to local inflammatory responses, organ dysfunction, disrupted cellular energetics, and deleterious effects on DNA, lipids, and proteins [1,2,3]. Pre-treatment with antioxidants can limit damage and inflammatory activation in animal experiments, but the responses are complex and model-dependent. Antioxidants can attenuate proliferation and thus hamper healing responses [4]. ROS scavengers limit the development of restenosis in animal models of vascular injury, but antioxidant therapy after myocardial infarction (MI) increases the rate of cardiac rupture [5].

The different effects of ROS and antioxidants reflect the large number of redox targets in a cell, the dynamics of redox signalling, and cell-specific redox networks [2]. In the cardiovascular system, redox signalling in endothelial cells appears to be of particular importance due to endothelial nitric oxide production and the unique bio-physical aspects of the endothelial niche [6]. Endothelial redox signalling has, therefore, been intensively studied regarding the interaction of superoxide and nitric oxide as well as the response of endothelial cells to hydrogen peroxide (H_2_O_2_) and its reaction with thiols [2].

Numerous studies have investigated H_2_O_2_ signalling by either adding the molecule to cells or changing the antioxidant defence. Both approaches are suboptimal. Extracellular administration results in uncertain intracellular concentrations and predominantly targets extracellular proteins. Antioxidant approaches are usually not suited to outcompete highly reactive thiols, and the high concentrations of antioxidants that must be administered to be effective have effects themselves. As an alternative, chemogenetic models to generate H_2_O_2_ in vivo have recently emerged. The most frequently used approach utilizes the enzyme D-amino acid oxidase (DAO), which catalyzes the conversion of D-amino acids to imino acids and ammonia, a reaction that produces H_2_O_2_ in equimolar concentrations as a by-product [7]. DAO has been utilized in a number of very interesting studies, particularly on cellular systems and the cardiovascular system of rodents [7,8,9,10,11,12,13,14,15,16].

Essentially, rodent studies illustrate that H_2_O_2_ production can be deleterious. This finding is interesting as it is well known that the effect of H_2_O_2_ is dose- and time-dependent. This makes studies on chemogenetic ROS production very difficult. The timing, the selection of the transgene, and the expression level of DAO and the concentration of the D-amino acid all impact the outcome of these studies. This not only results in an experimental bias towards the most deleterious effect but also requires confirmation. Here, we generated a tamoxifen-inducible, endothelial cell-specific knock-in mouse expressing the yeast DAO using the flox-stop-flox technique. Although DAO in this system was active, the effect on the cardiovascular system was modest and greatly dependent on the time point studied.

## 2. Materials and Methods

### 2.1. Generation of yDAO-HyPer7 Construct

The yDAO-HyPer7-NES construct under the control of the CAG promoter was designed in silico using SnapGene (5.3), with the yeast DAO homologue (DAO1 from Rhodotorula gracilis, ATCC 26217 [17]), which exhibits high activity. The fully synthetic construct was obtained from Eurofins (Ebersberg, Germany). In the plasmid, the coding sequence of the enzyme was fused to that of HyPer7, a sensitive fluorescent ratiometric H_2_O_2_ reporter protein, as reported previously by others [18]. The construct also contained a nuclear exporting sequence (NES). Promoter and coding sequence were separated by LoxP sites flanking three SV40 poly (A) signals (STOP). This allows gene expression after recombination using a Cre-recombinase. To test the function of the fully synthetic plasmid, we analyzed the ROS level through the HyPer7 construct or measured H_2_O_2_ by chemiluminescence. For this, HEK 293 cells were transfected with yDAO-HyPer7-NES and Cre-recombinase plasmids. HyPer7 was imaged in cells using a LSM510 Meta. yDAO-HyPer7-NES was excited sequentially via laser lines passing through 427/10 and 504/12 bandpass excitation filters, respectively. Emission was determined every 20 s using a 525/50 bandpass emission filter. After 5–10 images were acquired, D-Ala (10 mM) was added to the cells.

### 2.2. Chemiluminescence Assays

Chemiluminescence in response to luminol (100 µM)/horseradish peroxidase (HRP, 1 U/mL) was measured in a Berthold 6-channel luminometer (LB9505, Berthold, Wildbad, Germany). H_2_O_2_ production from HEK 293 cells (transfected with yDAO-HyPer7-NES and Cre-recombinase plasmids) or mLEC (mouse lung endothelial cells at 100,000) of ecDAO (Hipp11-Flox-Stop-Flox-yDAO-Cdh5-CreERT2^+/0^) mice and CTL littermates were initiated by addition of L-Alanine or D-Alanine (10 mM). PEG-catalase (500 U/mL) was used as indicated. All measurements were performed in HEPES-Tyrode buffer containing in mmol/L: 137 NaCl, 2.7 KCl, 0.5 MgCl_2_, 1.8 CaCl_2_, 5 glucose, 0.36 NaH_2_PO_4_, 10 HEPES. Chemiluminescence was expressed in arbitrary units.

### 2.3. Animal Procedure

The yDAO-HyPer7-NES construct contained specific overhangs for integration into the Hipp11 locus of mice. The murine Hipp11 locus is an active genome region that allows for the stable expression of large transgenes (safe harbour). The DAO-floxed mice (Hipp11-yDAO-HyPer7^tm1NH^) were generated in the Laboratory Animal Resource Center, University of Tsukuba, Japan, by means of CRISPR/Cas9-mediated homology-directed repair as previously described [19]. ecDAO mice were obtained by crossing Hipp11-yDAO-HyPer7^tm1NH^ mice with Cdh5-CreERT2 (Tg (Cdh5-CreERT2)^1Rha^) [20] mice (kindly provided by Ralf Adams, Münster, Germany). DAO expression was induced by providing tamoxifen with food (400 mg/kg, 10 days) when animals were at least 8 weeks old. A tamoxifen-free “wash out” period of at least 14 days after tamoxifen feeding was adhered to. Control animals (CTL) were defined as Hipp11-yDAO-HyPer7^flox/flox^-Cdh5-CreERT2^0/0^ littermates (i.e., no Cre expression) and were also treated with tamoxifen.

All animals had free access to chow and water in a specified pathogen-free facility with a 12 h day/12 h night cycle. All animal experiments were performed in accordance with the German animal protection law and were carried out after approval by the local authorities. Every mouse received an identification number for each experiment and the experimentor was blind to the genotype, sex, or treatment. Animal group sizes differ due to the number of littermates. Control and knock-in animals were studied in a paired fashion per experiment, and male as well as female animals were used.

### 2.4. Minimally Invasive Myocardial Infarction

A minimally invasive myocardial infarction (MI) method was employed to induce heart failure in ecDAO and CTL mice as described previously [21]. Briefly, the left anterior descending artery was first evaluated by ultrasound in B-mode imaging. To target the vessel, a micromanipulator-controlled monopolar needle was inserted into the chest. Coagulation of the coronary artery was achieved by high-frequency electricity using an electrosurgical unit. A successful MI was determined by the absence of blood flow distal of the occlusion (using Doppler imaging) and akinesia in the affected part. D-Ala (0.5 M) was added to drinking water one day prior to the surgery and up to 52 days post-MI. Cardiac function was assessed by echocardiography in B- and M-mode.

### 2.5. Vascular Reactivity Measurements

Isolated vascular contraction recordings were performed as previously described using isometric force transducers [22]. Briefly, the chambers were first filled with modified Krebs–Henseleit buffer, pH 7.4, which was constantly aerated with carbogen (95% O_2_ and 5% CO_2_). Vascular rings were pre-stretched in 0.2 g steps to a final force of 1 g. The Krebs–Henseleit buffer was replaced with an 80 mM KCl-enriched solution to induce vascular contraction. Once the constriction reached a plateau, KCl was washed out using the Krebs–Henseleit buffer. KCl-enriched buffer was added a second time to induce a maximal constriction that was set as the maximal contractile response for each ring. After washing, re-equilibration, and zeroing, phenylephrine was then added in cumulative doses ranging from 1 nmol/L to 10 μmol/L until the vascular ring was constricted to 80% of the maximal KCl constriction. Subsequently, acetylcholine was cumulatively added from 1 nmol/L to 10 μmol/L to determine the endothelium-dependent dilator response. Afterwards, a second dose–response curve was generated using the NO donor DETA NONOate (10 nmol/L–10 μmol/L) to determine endothelium-independent relaxation.

### 2.6. Isolation of Mouse Lung Endothelial Cells

Murine lung endothelial cells (mLECs) were isolated from freshly harvested lungs of ecDAO and control mice as described previously [23]. Briefly, the tissue was minced and digested by dispase II 5 U/mL (#04942078001, Roche, Mannheim, Germany) at 37 °C. After several washing steps, mLECs were separated magnetically using CD114-coated Dynabeads (MACS^®^, Miltenyi Biotec, Cologne, Germany) and cultured in D-MEM/F12 (#11039-047, Gibco, Karlsruhe, Germany) supplemented with 20% fetal calf serum, ECGF (#PB-ECGF-1 Heparin, 6 mg, from PeloBiotech, Planegg, Germany), and 0.5% Penicillin/Streptomycin. mLECs were used for the experiments between passages 3 and 4.

### 2.7. Western Blot Analysis

Mouse tissue, HEK 293 cells, or mLECs were lysed with a Triton-based buffer at pH 7.4, with the following concentrations in mmol/L: Tris-HCl (50), NaCl (150), sodium pyrophosphate (10), sodium fluoride (20), Triton X-100 (1%), sodium desoxycholate (0.5%), proteinase inhibitor mix, phenylmethylsulfonyl fluoride (1), orthovanadate (2), and okadaic acid (0.00001). Proteins (30 µg) were separated by SDS/PAGE, transferred by Western blot, and probed with the antibodies listed in Table 1. Western blot analyses were performed with an infrared-based detection system (Odyssey, Licor, Bad Homburg, Germany).

### 2.8. Echocardiography

Cardiovascular parameters were assessed with ultrasonography [24] using a Vevo3100 device (Toronto, Canada). Data analysis was performed using the Vevo LAB desktop software (Vevo LAB 5.8.1). Measurements were obtained from short-axis M-mode images and left common coronary artery diameter. 

### 2.9. Histology

Hearts from ecDAO mice and control littermates were freshly isolated, embedded in OCTTM (Tissue-Tek^®^), and frozen on dry ice. Frozen tissues were cut (8 µm) and placed on Superfrost^®^ slides. For the assessment of the cross-sectional area of cardiac myocytes, heart sections were stained with wheat germ agglutinin (WGA, Alexa Fluor 488 conjugate, #W11261, dilution 1:50, Life Technologies, Carlsbad, CA USA). For this, sections were fixed with precooled acetone for 30 min at 4 °C and dried for 20 min at RT. Sections were then incubated for 30 min at RT with blocking buffer (2% BSA, 0.2% Triton X-100 in PBS). Muscle fibres were stained for 40 min with Alexa-Fluor 488-WGA (2% BSA, 0.2% Triton X-100, 0.1% 1 mM CaCl_2_, WGA, wheat germ agglutinin, 1:50 in PBS) at RT. Nuclei were stained with DAPI (1:200). Images were captured from areas of transversely cut muscle fibres using a confocal microscope (LSM800) and analyzed with ZEN lite software (Zen 3.5, ZEN lite, Zeiss, Jena, Germany). The cross-sectional area of cardiomyocytes was determined using MATLAB software (MATLAB R2024a).

### 2.10. En Face Immunofluorescence 

The thoracic aorta was dissected and portioned into segments of 2 mm that were cut open transversally. Vessel segments were fixed in 4% PFA for 5 min followed by a permeabilization/blocking step with 1% BSA, 0.1% Triton X-100, and donkey serum for 4 h at RT. Next, the vessel segments were incubated with primary antibody against ZO-1 (1:200, Invitrogen, Waltham, MA, USA, #617300) at 4 °C overnight. The vessel segments were then washed three times with 0.1% Tween in PBS followed by two washing steps with only PBS for 5 min each and then incubated with respective secondary antibodies for 2 h. Vessels were mounted in Dako Fluorescence Mounting medium (Agilent Technologies Inc., Santa Clara, CA, USA), and fluorescence images were acquired with a Zeiss LSM800 laser scanning microscope (Carl Zeiss Microscopy GmbH, Jena, Germany).

### 2.11. Statistics

Data are expressed as the mean ± standard error of the mean. Calculations were performed with Prism 10.1.1. The latter was also used to test for normal distribution and similarity of variance. In the case of multiple testing, a Bonferroni correction or Tukey’s test was applied. For multiple-group comparisons, analysis of variance followed by post hoc testing was performed. Individual statistics were obtained by the Mann–Whitney test as indicated. *p* values of <0.05 were considered significant. Unless otherwise indicated, n indicates the number of individual mice or experiments.

## 3. Results

### 3.1. Generation of an Inducible, Endothelial Cell-Specific Knock-In Mouse of DAO

**Figure 1:**
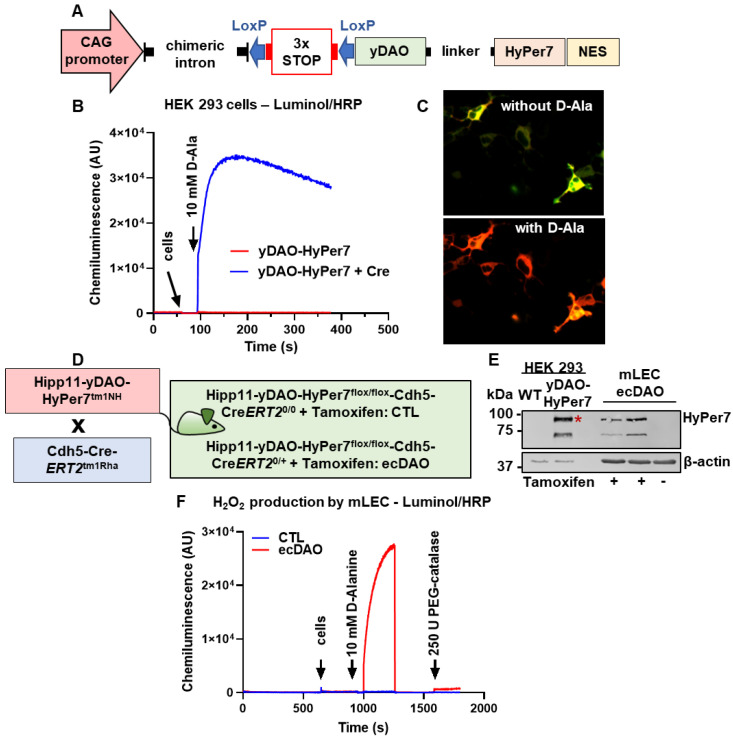
Generation and validation of an inducible, endothelial cell-specific chemogenetic mouse model of yDAO to generate H_2_O_2_. (**A**): Design of the yDAO-HyPer7-NES construct. H_2_O_2_ production as measured by luminol/HRP (**B**) or HyPer ratiometric fluorescence (**C**) in HEK 293 cells double transfected with plasmids coding for yDAO-HyPer7-NES and a Cre-recombinase plasmid. (**D**): Generation of a knock-in, tamoxifen-inducible, endothelial cell-specific mouse model of yDAO by crossing Hipp11-DAO-HyPer7^tm1NH^ with Chd5-Cre-ERT2 (Tg (Cdh5-CreERT2)^1Rha^) mice. Mice fed with tamoxifen to induce yDAO-HyPer7-NES expression specifically in endothelial cells are denoted as ecDAO. Control mice (CTL) are those that do not express Cre-recombinase but receive tamoxifen. (**E**): Western blotting for HyPer7 from lung endothelial cells (mLECs) isolated from ecDAO mice (without or with tamoxifen) as indicated. WT denotes HEK cells without transfection. * indicates the band observed for HyPer7. (**F**): H_2_O_2_ measurements using luminol/HRP from mLEC as indicated.

To develop a chemogenetic approach that allows for the controlled production of intracellular H_2_O_2_, we designed a construct under the control of the CAG promoter containing the yDAO-HyPer7-NES (Figure 1A) in silico. The promoter and coding sequence were separated by three SV40 poly (A) signals (STOP) that were flanked by one LoxP site each. This allows gene expression after recombination using a Cre-recombinase. To test the function of the construct, overexpression studies were performed in HEK 293 cells. The construct was transfected transiently with or without an expression plasmid for Cre-recombinase. H_2_O_2_ was determined by luminol/HRP-coupled chemiluminescence. The addition of 10 mM D-Ala resulted in strong formation of H_2_O_2_ in HEK 293 only when yDAO-HyPer7-NES and Cre-recombinase were co-transfected (Figure 1B). No signal was observed in cells expressing yDAO-HyPer7-NES alone. yDAO was fused to HyPer7, a pH-stable and sensitive fluorescent ratiometric H_2_O_2_ reporter protein [18]. After oxidation, the excitation/absorption spectrum of HyPer7 is changed in a ratiometric manner, with a decrease at 400 nm and an increase in the 499 nm absorbance peak. Similarly to the luminol signal, the ratiometric signal of HyPer7 in HEK 293 cells double transfected with Cre-recombinase and yDAO-HyPer7-NES also confirmed H_2_O_2_ production upon the addition of D-Ala (Figure 1C). Together, these results indicate that the developed construct was functional, and yDAO-HyPer7-NES expression was controlled by Cre-recombinase.

To generate yDAO-HyPer7-NES knock-in mice, wild-type mice with the C57Bl6J background were used. The construct was integrated into the Hipp11 locus of mice by means of CRISPR/Cas9-mediated homology-directed repair, as previously described [19]. Hipp11-flox-STOP-flox-yDAO-HyPer7-NES mice were crossed with Cdh5-CreERT2 mice to generate an endothelial cell-specific, tamoxifen-inducible knock-in mouse of yDAO (ecDAO). Five mouse lines were generated, and the one showing the highest H_2_O_2_ production, as measured by luminol/HRP in isolated mouse lung endothelial cells, was selected. Control mice (CTL) harbour the Hipp11-yDAO-HyPer7-NES but do not express Cre-recombinase (Figure 1D). Both mouse lines received tamoxifen for 10 days, followed by a wash out time of 15 days. Mice expressing Cre-recombinase that received no tamoxifen feeding were used as negative controls for the validation of the mouse line. After Cre-recombinase activation by tamoxifen, mouse lung endothelial cells were isolated and the expression of the construct was assessed by Western blotting using an antibody against GFP (green fluorescent protein) that recognizes HyPer7.

HyPer7 was only detected in Cre-recombinase-positive mice that received tamoxifen, demonstrating that the construct was expressed in vivo (Figure 1E). Next, we measured H_2_O_2_ with luminol/HRP in isolated mLECs from CTL and ecDAO mice that received tamoxifen. Upon D-Ala (10 mM) addition, a strong increase in H_2_O_2_ production by mLECs was detected in cells harvested from mice expressing ecDAO but not in those obtained from CTL mice. As expected, the signal was sensitive to PEG-catalase (250 U), confirming direct H_2_O_2_ production (Figure 1F). Collectively, these data illustrate that we succeeded in generating a mouse line with targeted endothelial expression of yDAO upon tamoxifen treatment.

### 3.2. Acute Chemogenetic Generation of H_2_O_2_ by Endothelial Cells Decreases Vascular Tone

**Figure 2:**
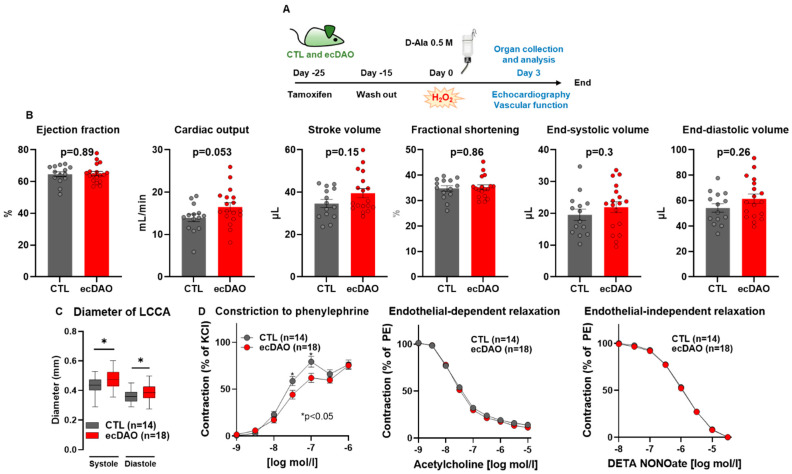
Acute chemogenetic generation of H_2_O_2_ by endothelial cells does not affect cardiac function but reduces vascular constrictor function in mice. (**A**): Schematic representation of the experimental design. The knock-in of yDAO-HyPer7-NES was induced by tamoxifen chow (400 mg/kg) for 10 days, followed by a wash out time of two weeks. CTL and ecDAO mice received D-Ala (0.5 M) in drinking water for three days. Cardiac function was measured with echocardiography, and the parameters are shown in (**B**). The diameter of the left common carotid artery was measured with ultrasound Vevo3100 using the vascular package (**C**). * *p* < 0.05, Tukey’s test. Vascular function was assessed with organ chamber experiments as indicated (**D**). * *p* < 0.05, two-way ANOVA for repeated measurements.

In order to characterize the ecDAO mice, an early and a late time point were chosen to determine cardiac function and other potential parameters. For the short-time scenario, Cre-recombinase was activated in ecDAO mice with tamoxifen, and after the wash out period, CTL and ecDAO mice both received D-Ala (0.5 M) with drinking water for three days. Cardiac function was monitored by echocardiography, and vascular function was assessed in vivo by carotid artery measurements and in isolated aortic rings using isometric tension recordings (Figure 2A).

The acute generation of H_2_O_2_ in endothelial cells did not have a strong effect on cardiac function. Interestingly, there was, however, a strong trend (*p* = 0.053) towards an increase in cardiac output in response to H_2_O_2_ (Figure 2B). Also, in vivo, vascular tone was reduced in this group, as evidenced by the greater diameter of the left common carotid artery (LCCA, in vivo ultrasound measurements, Figure 2C). Both parameters together may suggest that increased endothelial H_2_O_2_ attenuates vascular tone and stiffness. Interestingly, ecDAO activation also resulted in a decreased sensitivity to the alpha-adrenergic constriction of the aorta, which would be compatible with this notion (Figure 2C). In contrast, endothelium-dependent and independent relaxation was similar between both groups. Collectively, the data suggest that ecDAO-derived H_2_O_2_ attenuates vascular tone. This finding is compatible with the idea of H_2_O_2_ being an acute vasodilator at low concentrations [25].

### 3.3. Prolonged Chemogenetic Generation of H_2_O_2_ by Endothelial Cells Induces Cardiovascular Dysfunction in Mice

**Figure 3:**
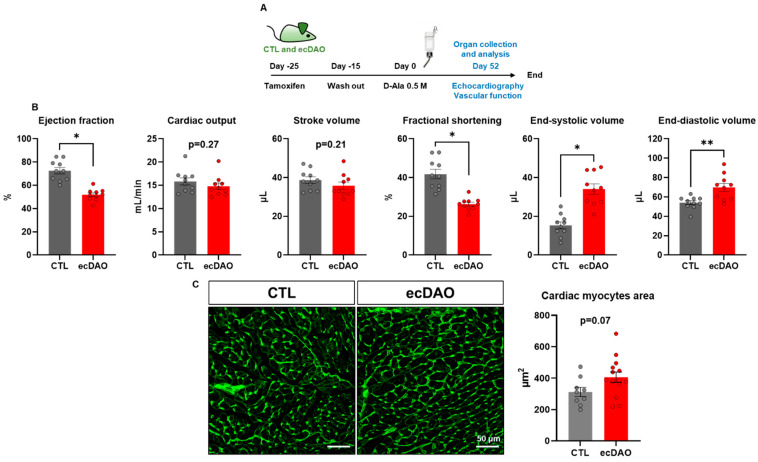
Prolonged chemogenetic generation of H_2_O_2_ by endothelial cells induces cardiac remodelling in mice. (**A**): Schematic representation of the experimental design. The knock-in of yDAO-HyPer7-NES was induced by tamoxifen chow (400 mg/kg) for 10 days, followed by a wash out time of two weeks. CTL and ecDAO mice received D-Ala (0.5 M) in drinking water for 52 days. Cardiac function was measured with echocardiography, and the parameters are shown in (**B**). * *p* < 0.05, ** *p* < 0.01, Mann–Whitney test. (**C**): Area of cardiac myocytes as determined by wheat germ agglutinin (WGA) staining. n ≥ 9 scale bar: 50 µm. *p* = 0.07, Mann–Whitney test.

To study a late time point, the effects of 52 days of D-Ala on cardiovascular function in CTL and ecDAO mice were assessed (Figure 3A). Prolonged chemogenetic generation of H_2_O_2_ by endothelial cells decreased the cardiac ejection fraction and fractional shortening. Moreover, it increased end-systolic and end-diastolic volume (Figure 3B). Comparing the different parameters, it appeared that the effect on the dynamic aspects of contractile function (i.e., EF, FS, end-systolic volume) changed more strongly than the cardiac structural parameters (end-diastolic volume, myocyte area). This combination would therefore suggest cardiac systolic dysfunction. Differences in cardiac systolic function can be the consequence of a primary myocyte dysfunction. To investigate this, the area of cardiac myocytes was estimated through WGA staining, which revealed only a non-significant trend towards an increased area of cardiac myocytes in ecDAO mice as compared to CTL mice (Figure 3C).

**Figure 4:**
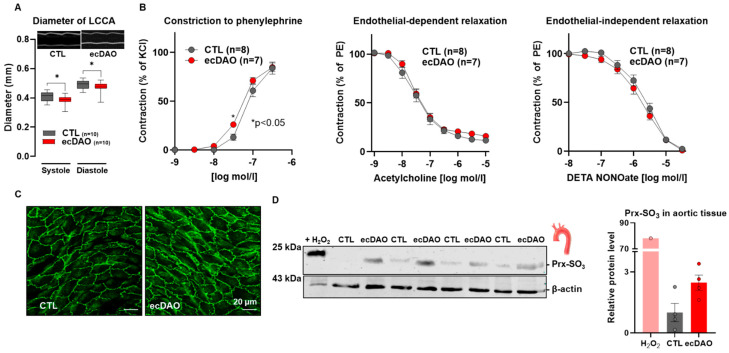
Prolonged chemogenetic generation of H_2_O_2_ by endothelial cells induces vascular dysfunction and increases protein oxidation in mice. (**A**): Diameter of the left common carotid artery (ultrasound Vevo3100 using the vascular package). * *p* < 0.05, Tukey’s test. (**B**): Vascular function as assessed with organ chamber experiments as indicated. * *p* < 0.05, two-way ANOVA for repeated measurements. (**C**): Endothelial integrity as shown by immunofluorescence for the tight junction protein ZO-1. (**D**): Western blotting for peroxiredoxin-SO_3_ from aortic tissue isolated from CTL and ecDAO mice that received D-Ala in drinking water for 52 days (experimental design represented in Figure 3A). Quantification by densitometry with bands normalized to β-actin. Positive controls are mLECs isolated from CTL mice treated with H_2_O_2_ (300 µM, 15 min).

Increased peripheral resistance and stiffness are parameters that can also underlie cardiac systolic dysfunction. To investigate this aspect, the diameter of the LCCA was measured by ultrasonography in vivo. Interestingly, upon chronic production of H_2_O_2_ by endothelial cells, the diameter of the LCCA of the ecDAO mice was significantly smaller as compared to the CTL mice (Figure 4A). A similar effect was also observed in isolated aortic segments. There was a significant increase in the aortic vasoconstrictor response of ecDAO mice to phenylephrine as compared to the CTL mice. Similarly to in the short-term group, endothelium-dependent and independent relaxation was identical between the groups (Figure 4B). Overall, prolonged chemogeneration of H_2_O_2_ by endothelial cells leads to cardiac remodelling, which might result from vascular stiffening or increased peripheral resistance.

### 3.4. Prolonged Generation of H_2_O_2_ by Endothelial Cells Does Not Affect EC Integrity but Increases Protein Oxidation

Redox signalling mediated by H_2_O_2_ can impact cell proliferation, apoptosis, and senescence and therefore endothelial integrity [1]. To investigate this aspect, aortic en face staining for the tight junction protein ZO-1 was performed, but this parameter was not affected by prolonged H_2_O_2_ generation (Figure 4C).

The prolonged effects of H_2_O_2_ can be mediated by overoxidation of thiol residues like sulfinic (SO_2_) and sulfonic acid (SO_3_) in proteins [26]. Peroxiredoxins (Prx) are well-known sensitive targets of H_2_O_2_ that can be reversibly and irreversibly oxidized [27]. Prx-SO_3_ has been used as a marker for protein overoxidation by H_2_O_2_. In line with this notion, Western blot analysis of whole aortic tissue from CTL and ecDAO mice that received D-Ala for 52 days was performed. Interestingly, there was an increase in Prx-SO_3_ adducts in the aortic tissue of the ecDAO mice, whereas they were hardly detected in the aortae of the CTL mice.

### 3.5. In Vivo Chemogenetic Generation of H_2_O_2_ in Endothelial Cells Does Not Affect the Outcome After Myocardial Infarction (MI)

As the present data suggest that ecDAO is active in the present model and affects cardiovascular function, it was hypothesized that additional stress may potentiate the effects. To study this aspect, mice were subjected to minimally invasive MI.

**Figure 5:**
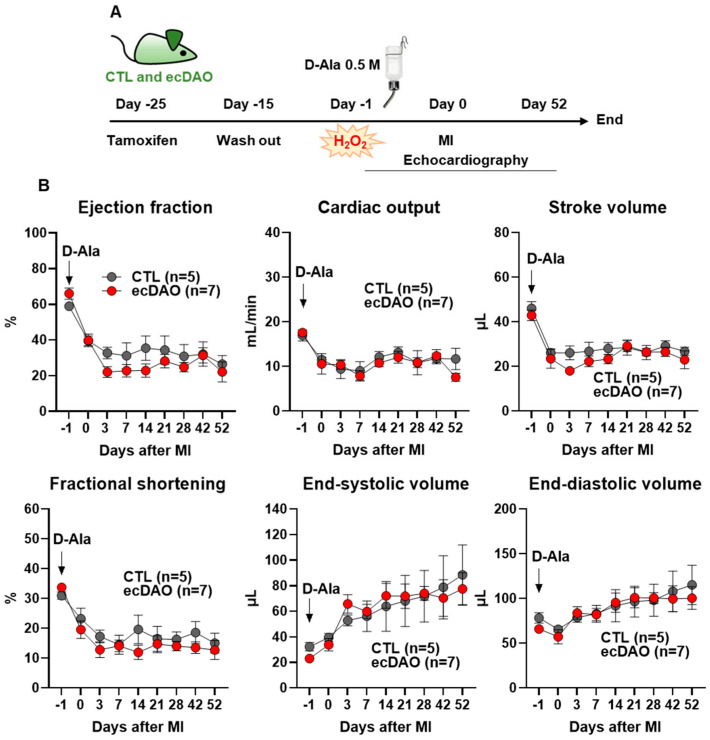
In vivo chemogenetic generation of H_2_O_2_ in endothelial cells does not affect the outcome of myocardial infarction. (**A**): Schematic representation of the experimental design for the minimally invasive myocardial infarction model using CTL and ecDAO mice. The knock-in of yDAO-HyPer7-NES was induced by tamoxifen chow (400 mg/kg) for 10 days, followed by a wash out time of two weeks. Mice received D-Ala (0.5 M) in drinking water from one day before the myocardial infarction until day 52. Cardiac function was monitored with echocardiography throughout the experiment, and the parameters are shown in (**B**) as indicated.

After the induction of Cre-recombinase with tamoxifen followed by the wash out period, the mice received D-Ala (0.5 M) in drinking water one day before the MI and continued to receive it for 52 days. Cardiac function was monitored by echocardiography (Figure 5A).

Immediately after the MI, the ejection fraction, cardiac output, stroke volume, and fractional shortening decreased in both strains, with a maximal effect 3–7 days post MI (Figure 5B). Conversely, end-systolic and diastolic volume continuously increased post MI. However, there was no significant difference in all cardiac parameters between the CTL and ecDAO mice. Thus, further histological analyses have not been performed. These data suggest that the effects of DAO-dependent H_2_O_2_ production are so moderate that they are overruled by a stronger stressor, like MI.

## 4. Discussion

In this study, an inducible, endothelial cell-specific chemogenetic approach to studying the effect of redox signalling in cardiovascular function in vivo was set up. An yDAO-HyPer7-NES construct harbouring STOP sites flanked by LoxP sites (flox) was developed, which allowed for a tamoxifen-inducible knock-in expression of the construct when co-expressed with Cre-recombinase. The overexpression experiments in HEK293 cells demonstrate the correct function of the construct, as determined by the documentation of H_2_O_2_ production as well as changes in the ratiometric signal of HyPer7. The construct contained overhangs to allow for its integration into the Hipp11 locus, which is an advantage as it facilitates the integration of large genes at a ‘safe harbour’ [19]. This strategy is advantageous over the ROSA26 locus, which, despite its broad use, has inherent instability and is already excessively used for several Cre-lines [28]. Hipp11-yDAO-HyPer7^flox/flox^-Cdh5-CreERT2 mice (^0/0^ or ^0/+^ for Cre-recombinase) were generated through crossing with the Cdh5-CreERT2 endothelial Cre-driver. The in vivo induction of yDAO-HyPer7-NES through tamoxifen feeding was also achieved, as demonstrated by Western blotting for GFP, which detects HyPer7, in isolated mLECs. Moreover, the mLECs generated H_2_O_2_ upon D-Ala administration, as detected by chemiluminescence measurements using luminol/HRP. Unfortunately, there is no antibody against the yeast DAO, and considering that DAO in the present model is expressed specifically in endothelial cells, the number of endothelial cells in the arteries is a limiting factor for the detection of HyPer7. Therefore, successful knock-in in ecDAO mouse tissue was also confirmed using Western blotting for HyPer7 in lung tissue, which is rich in endothelial cells.

The central finding obtained with this model is that the acute endothelial generation of H_2_O_2_ induces a reduction in vascular tone or stiffness, whereas prolonged H_2_O_2_ instead induces an increase in stiffness and tone. These findings fit well with the established concept that acute stimulation with H_2_O_2_ increases protein kinase G signalling [29,30] and may induce nitric oxide synthase [31,32] and that Nox4, which is an important source of low concentrations of H_2_O_2_ in the vessel wall, maintains a physiological dilator tone [23,33]. Moreover, H_2_O_2_, through controlling NRF2, also positively impacts vascular H_2_S production, which also reduces vascular stiffness [34]. Data from mice overexpressing catalase suggest that prolonged H_2_O_2_ at higher concentrations induces vascular dysfunction [35] as a consequence of the induction of vasoconstrictor prostaglandins and calcium increasing the stimulation of superoxide production [36], alongside numerous other mechanisms [37,38]. However, in the presence of a strong stressor or a much higher concentrations of ROS, the cardiovascular effects of DAO-dependent H_2_O_2_ production are moderate, as demonstrated here through MI.

What does the present study have to offer compared to other published studies on chemogenetic H_2_O_2_ proteins? There is no doubt that these studies have been highly interesting in linking H_2_O_2_ to several forms of dysfunction. The first model was developed in 2018, in which rats were infected with adeno-associated virus serotype 9 (AAV9) containing yDAO-HyPer under the control of the cardiac-specific TnT (cardiac troponin) promoter (DAAO-TGCar) [15]. Upon D-Ala treatment, DAAO-TGCar animals developed cardiac oxidative stress and showed signs of dilated cardiomyopathy with a decreased ejection fraction and an increased chamber size. Despite the pathological phenotype, this model is prone to insertional mutagenesis and off-target effects associated with viral vectors. To overcome these limitations, the first flox-STOP-flox-yDAO-HyPer was generated in mice (DAAO-TGLoxP), and they were crossed with constitutive Cdh5-Cre promoter mice (DAAO-TGCdh5). This led to a phenotype of neurovascular oxidative stress upon D-Ala administration through drinking water. The mice showed severe sensory ataxia, hypertrophic cardiomyopathy, and an increase in the ejection fraction and wall thickness [9]. Such a phenotype could be partially explained by the fact that in dorsal root ganglia the Chd5 promoter is transiently active in fetal development, which could be sufficient to activate DAO expression by removing the STOP codon in this cell type. Interestingly, the cardiac phenotype observed in DAAO-TGCdh5 mice was not observed in DAAO-TGLoxP mice crossed with another endothelial promoter, the Tie2-Cre-line from Jackson’s lab (DAAO-TGTie2).

Here, we show that the inducible expression of yDAO, specifically in endothelial cells using Cdh5-Cre-ERT2, recapitulates partially the cardiac phenotype observed in other DAO lines. Chronic exposure to D-Ala and consequently H_2_O_2_ production by endothelial cells in ecDAO mice decreased the ejection fraction and fractional shortening. Conversely, the end-systolic and end-diastolic volumes, as well as the size of cardiac myocytes, were increased in the ecDAO mice as compared to the CTL mice. Such alterations suggest cardiac remodelling, which could be a consequence of an increase in vascular tone or stiffness. In fact, we observed a decrease in the diameter of the LCCA in vivo and increased vessel constriction upon exposure to phenylephrine in vessels isolated from the ecDAO mice as compared to those from the CTL mice (52 days of D-Ala). Interestingly, the acute chemogeneration of H_2_O_2_ by endothelial cells (3 days of D-Ala) slightly improved vascular function without affecting cardiac function. H_2_O_2_ is well known to promote eNOS expression and activity [39,40]. In cultured EA.hy926 human endothelial cells, chemogenetic H_2_O_2_ generation by DAO showed differential signalling according to its subcellular compartment [11]. However, H_2_O_2_ is thought to diffuse across membranes and through aquaporins [41,42]; therefore, its dynamics and effects on eNOS in vivo might be different to those in cultured cells. Given that the vascular effect of acute chemogenetic H_2_O_2_ generation by endothelial cells in the ecDAO mice as compared to the CTL mice was rather mild, we did not further investigate eNOS activation as a potential molecular mechanism. Nevertheless, these short-term effects can only be mediated by H_2_O_2_ given that the patho- and physiological effects of ammonia occur first at mM concentrations but at µM concentrations for H_2_O_2_ [15].

The fact that prolonged chemogenetic H_2_O_2_ generation by endothelial cells increased peroxiredoxin overoxidation in aortic tissue was a striking finding. Moreover, endothelial cell integrity was not affected despite the overoxidation of peroxiredoxins in the aortic tissue. The specific cell type in which such overoxidation occurred as well as the target proteins could not be disclosed due to technical limitations hindering us from isolating different cell types in sufficient amounts while maintaining their redox properties. Nevertheless, the observation that endothelial cells indeed harbour a particular redox signature is interesting for future studies.

*Limitations of the study:* The expression of DAO specifically in the endothelial cells of arteries could not be demonstrated as there is no antibody against the yeast DAO and the number of endothelial cells in the arteries is a limiting factor for the detection of HyPer7 using an anti-GFP antibody. 

Although HyPer7 was readily detected within the yDAO-HyPer7 fusion protein by Western blot, it was not possible to obtain significant fluorescence from the construct in mouse tissue. Potentially, nitric oxide produced by endothelial cells nitrosates the cysteine residues in HyPer7 or some other modifications inactivate it.

## 5. Conclusions

In conclusion, we generated an inducible, endothelial cell-specific (Cdh5-Cre-ERT2) chemogenetic mouse model to generate H_2_O_2_ in vivo. The chronic generation of H_2_O_2_ by endothelial cells induced cardiac remodelling and vascular dysfunction, which were associated with an increase in the vascular overoxidation of peroxiredoxins.

## Figures and Tables

**Table 1:** List of antibodies.

Name	Host	Manufacturer	Catalogue Number
β-Actin	Mouse	Sigma-Aldrich Taufkirchen, Germany	A1978
GFP	Rabbit	Cell Signaling Technology, Leiden, Netherlands	2956
Prx-SO_3_	Rabbit	Abcam, Cambridge, UK	Ab16830

## Data Availability

Protocols will be provided by Flávia Rezende (rezende@vrc.uni-frankfurt.de) upon request. Mouse lines will be shared under a Materials Transfer Agreement.
